# Non-Hodgkin lymphoma mimicking cholangiocarcinoma

**DOI:** 10.1590/0100-3984.2017.0134

**Published:** 2019

**Authors:** Gustavo Gomes Mendes, Leonardo Verza, Tércia Neves, Eduardo Nóbrega Pereira Lima, Rubens Chojniak

**Affiliations:** 1 A.C.Camargo Cancer Center, São Paulo, SP, Brazil.

**Figure 1:**
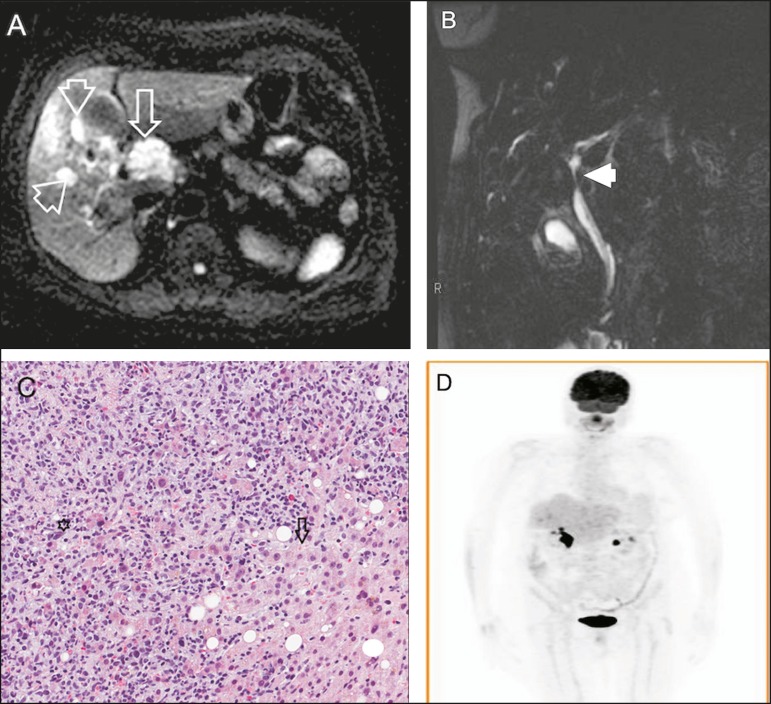
**A:** Axial diffusion-weighted MRI (b: 600 s/m2) showing a 4-cm expansive lesion in hepatic hilum (arrow) and small tumors focus around (arrowheads) characterized by signs of diffusion restriction, a common feature but not specific for lymphoma. **B:** Coronal strongly T2-weighted cholangioresonance showing stenosis of the common hepatic (arrowhead), related to extrinsic compression. This pattern is very similar to cholangiocarcinoma, therefore, being the main differential diagnosis. **C:** Hematoxylin-eosin staining (20×) showing lymphoid neoplasm (asterisk) characterized as diffuse large B-cell lymphoma (CD20 positive) infiltrating extensively hepatic parenchyma (arrow). **D:** Coronal fluorodeoxyglucose PET/CT showing no uptake in hepatic hilum and retroperitoneum six months after combined chemotherapy.

Dear Editor,

A 74-year-old white female presented with diffuse abdominal pain, jaundice, choluria, and acholia. Laboratory tests showed elevated levels of canalicular membrane enzymes. The results of a complete blood count were normal, as were serum alpha-fetoprotein levels, and a serological test for hepatitis was negative. Magnetic resonance imaging (MRI) showed a lesion in the hepatic hilum (Figure 1A), promoting common bile duct obstruction (Figure 1B) and retroperitoneal lymph node enlargement. A lymph node biopsy was negative for malignancy, and a liver biopsy showed a diffuse large B-cell lymphoma infiltrating the liver parenchyma (Figure 1C), together with positivity for markers of Epstein-Barr virus. A positron emission tomography/computed tomography (PET/CT) study, conducted for staging, showed fluorodeoxyglucose uptake in the retroperitoneum. The patient was started on combined chemotherapy with rituximab, cyclophosphamide, adriamycin, vincristine, and prednisone. Another PET/CT study, conducted six months later, showed no evidence of disease (Figure 1D).

Hepatic lymphomas are classified as primary or secondary^(^^1^^)^. Secondary lymphomas are due to disseminated lymphoproliferative disease, and the reported incidence of secondary non-Hodgkin lymphoma is 16-22%. Primary lymphomas are rare, accounting for only 0.01% of all cases of non-Hodgkin lymphoma^(^^1^^,^^2^^)^.

The diagnostic criteria for primary hepatic lymphoma vary in the literature, the most widely used criteria being those proposed by Lei et al.^(^^1^^)^: absence of distal lymphadenopathy; absence of bone marrow infiltration or peripheral blood leukocytosis; and laboratory abnormalities related to liver involvement. Caccamo et al.^(^^2^^)^ included the absence of extrahepatic disease for at least six months after diagnosis. However, some authors have also classified patients with associated regional lymph node disease, splenomegaly, and bone marrow infiltration as having primary hepatic lymphoma, those features being considered indicative of regional extrahepatic evolution^(^^3^^-^^5^^)^. The etiology is poorly understood, and there have been reports of cases related to viral infections, such as HIV infection, Epstein-Barr virus infection, and hepatitis, as well as to cirrhosis, prior chemotherapy, and primary biliary cirrhosis^(^^1^^,^^6^^)^. The usual symptoms are those associated with involvement of the liver parenchyma, such as jaundice, abdominal pain, and hepatomegaly^(^^1^^)^, similar to those of primary lymphoma described in the literature^(^^1^^,^^3^^,^^7^^)^ and well characterized in our patient due to a common bile duct obstruction. Fever, weight loss, and night sweats, also known as "B symptoms", may be present but are not the rule^(^^1^^)^. Elevated levels of canalicular enzymes is a common laboratory finding^(^^1^^)^. 

The imaging features of primary lymphomas are nonspecific and may be similar to those of other more common liver tumors, such as cholangiocarcinoma^(^^8^^)^. High-grade lymphomas usually show restricted diffusion on MRI, similar to what was observed in our patient but also seen in some infectious processes, such as abscess and fungal infections, in patients who are immunocompromised^(^^8^^)^, which our patient was not.

According to the current criteria in the literature, our patient had aspects indicative of primary and secondary hepatic lymphoma. Although the biopsy of an enlarged retroperitoneal lymph node was negative for malignancy, the PET/CT scan showed retroperitoneal fluorodeoxyglucose uptake. Although the diagnosis can be made through needle biopsy, it is often made after surgical resection. The standard treatment is systemic chemotherapy^(^^6^^,^^8^^)^.
